# Vaccination prevents severe COVID-19 outcome in patients with neutralizing type 1 interferon autoantibodies

**DOI:** 10.1016/j.isci.2023.107084

**Published:** 2023-06-09

**Authors:** Anette S.B. Wolff, Lena Hansen, Marianne Aa. Grytaas, Bergithe E. Oftedal, Lars Breivik, Fan Zhou, Karl Ove Hufthammer, Thea Sjøgren, Jan Stefan Olofsson, Mai Chi Trieu, Anthony Meager, Anders P. Jørgensen, Kari Lima, Kristin Greve-Isdahl Mohn, Nina Langeland, Rebecca Jane Cox, Eystein S. Husebye

**Affiliations:** 1 Department of Medicine, Haukeland University Hospital, 5021 Bergen, Norway; 2 Department of Clinical Science, University of Bergen, 5021 Bergen, Norway; 3 Influenza Centre, Department of Clinical Science, University of Bergen, 5021 Bergen, Norway; 4 Centre for Clinical Research, Haukeland University Hospital, 5021 Bergen, Norway; 5 Biotherapeutics Group, The National Institute for Biological Standards and Control, South Mimms, Potters Bar EN6 3QG, UK; 6 Department of Endocrinology, Oslo University Hospital, 0372 Oslo, Norway; 7 Department of Paediatric Medicine, Oslo University Hospital, 0372 Oslo, Norway; 8 Department of Endocrinology, Akershus University Hospital, 1478 Lørenskog, Norway; 9 Department of Microbiology, Haukeland University Hospital, 5021 Bergen, Norway

**Keywords:** Health sciences, Biological sciences, Immunology, Immune response

## Abstract

A hallmark of patients with autoimmune polyendocrine syndrome type 1 (APS-1) is serological neutralizing autoantibodies against type 1 interferons (IFN-I). The presence of these antibodies has been associated with severe course of COVID-19. The aims of this study were to investigate SARS-CoV-2 vaccine tolerability and immune responses in a large cohort of patients with APS-1 (N = 33) and how these vaccinated patients coped with subsequent infections. We report that adult patients with APS-1 were able to mount adequate SARS-CoV-2 spike-specific antibody responses after vaccination and observed no signs of decreased tolerability. Compared with age- and gender-matched healthy controls, patients with APS-1 had considerably lower peak antibody responses resembling elderly persons, but antibody decline was more rapid in the elderly. We demonstrate that vaccination protected patients with APS-1 from severe illness when infected with SARS-CoV-2 virus, overriding the systemic danger of IFN-I autoantibodies observed in previous studies.

## Highlights

• Patients with APS-1 had a mild response to COVID-19 vaccination with increases in spike IgG
• Patients with APS-1 did not mirror the profile of vaccine responses of elderly people
• Different COVID-19 vaccines were well tolerated by patients with APS-1
• Vaccination prevents severe COVID-19 outcomes in patients with IFN-I antibodies

## Graphical abstract

**Figure:**
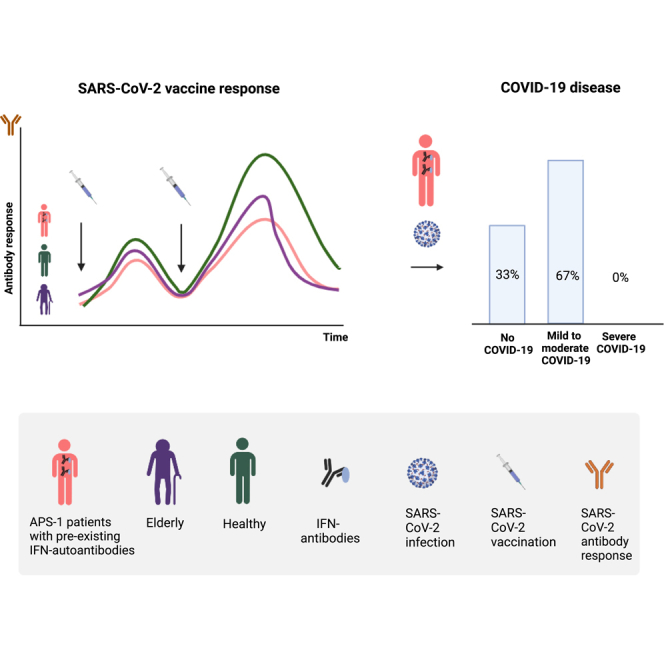

## Introduction

The severe acute respiratory syndrome coronavirus 2 (SARS-CoV-2) (COVID-19) pandemic has been one the largest health threats in modern time. Over 662 million people have been affected so far, and official data report more than 6.7 million deaths worldwide.^1^ As with influenza, several risk factors for severe disease have been identified, including age, obesity, and pre-existing disorders (reviewed in the study by Leretter et al.^2^). Among the reported risk comorbidities are autoimmune disorders and immune deficiencies, in particular rare inborn errors of immunity (IEI).^2^^,^^3^^,^^4^ One explanation for the increased risk is insufficient type 1 and type 2 interferon (IFN-I and IFN-II) production, which is essential for the antiviral response,^5^^,^^6^^,^^7^^,^^8^^,^^9^^,^^10^ including monogenic disorders of genes involved in IFN-synthesis or the presence of neutralizing IFN-autoantibodies. Examples of the latter are myasthenia gravis,^11^ autoimmune polyendocrine syndrome type I (APS-1),^12^ immunodysregulation, polyendocrinopathy, enteropathy, X-linked,^13^ and *RAG* mutation^14^ syndromes. Surprisingly, it was also recently discovered that antibodies against IFN-I were quite common (4%) among persons over 70 years of age.^15^ The presence of these antibodies correlates with severe COVID-19 illness, especially lung disease and fatal outcome.^15^ Patients with APS-1, who have germline mutations in the *autoimmune regulator (AIRE)* gene, are of specific interest as almost all of these individuals have preexisting, extremely high levels of neutralizing antibodies against IFN-I. Lack of AIRE function leads to both autoimmune and immune deficiency components.^16^^,^^17^^,^^18^ Indeed, several case studies have reported severe outcome and increased mortality for patients with APS-1 infected in the early waves of the COVID-19 pandemic before vaccination became widespread.^5^^,^^6^^,^^19^^,^^20^^,^^21^

We hypothesized that IFN-I antibodies interfere with the adaptive immune response against SARS-CoV-2, although this was recently challenged by Sokal and coworkers who found that patients with APS-1 (at least young individuals) have adequate anti-SARS-CoV-2 humoral vaccine responses.^22^ Hence, knowledge of the potential association of IFN-I antibodies and COVID-19 severity is still lacking. Previous studies were mostly conducted on small groups of unvaccinated patients early in the pandemic, infected with the Wuhan, Alpha (B.1.1.7) and Delta variants of concern (B.1.617.2), whereas we are now able to study the highly infectious Omicron variants (B.1.1.529, BA.1, BA.1.1, BA.2, BA.3, BA.4, and BA.5 lineages) in a vaccinated cohort.

Taking advantage of one of the world’s largest national registries and biobanks on patients with APS-1 (the Norwegian registry and biobank for organ-specific autoimmune disorders), we have here followed these patients longitudinally pre- and post-vaccination to investigate the tolerability and humoral specific immune responses to SARS-CoV-2 vaccination. We have also addressed in a cross-sectional design how SARS-CoV-2 infections affect vaccinated patients with APS-1.

## Results

### COVID-19 vaccination is well tolerated in patients with APS-1

**Figure 1:**
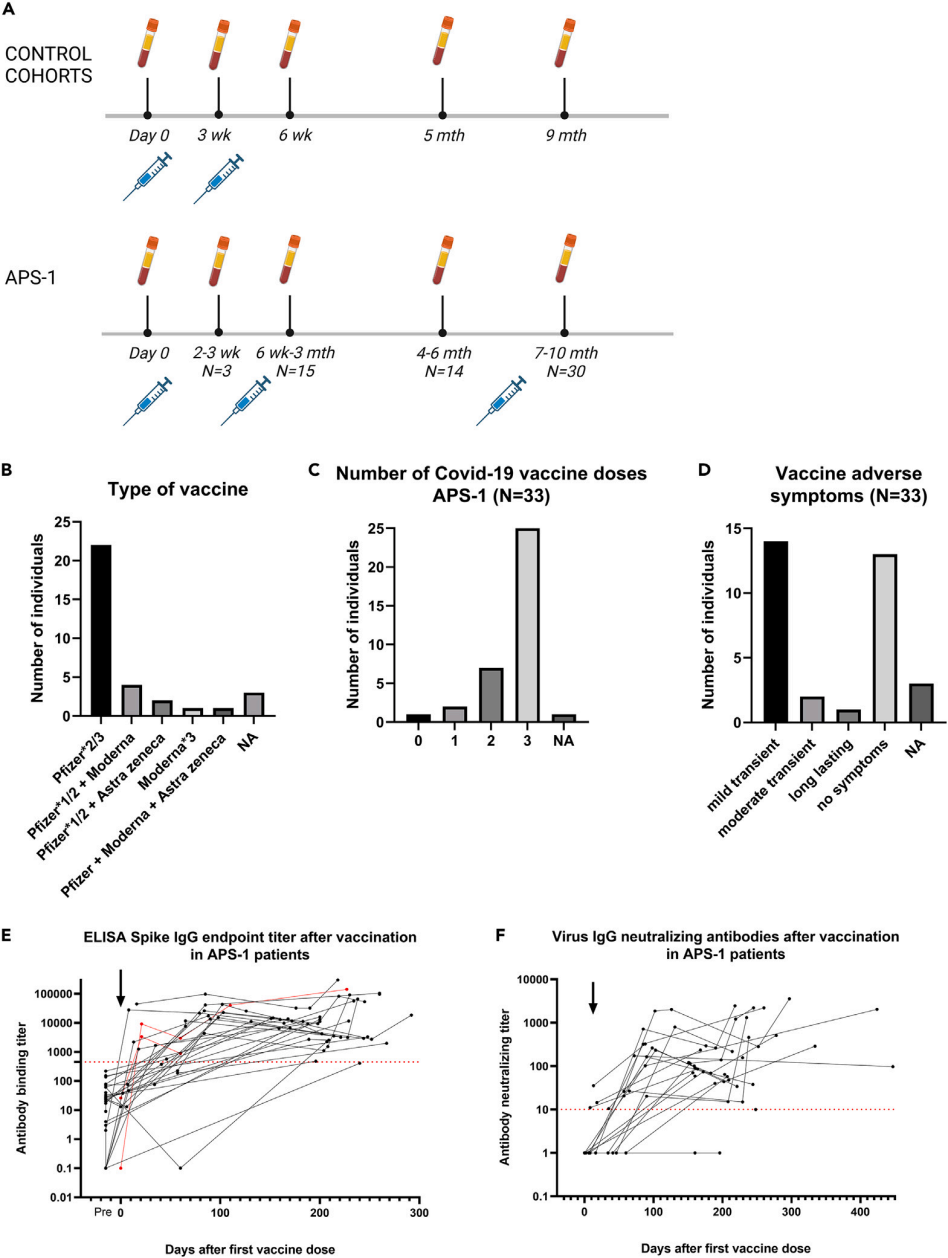
APS-1 SARS-CoV-2 vaccine study (A) Vaccine and sampling design for patients with APS-1 and age- and gender-matched and elderly healthy control groups. For patients, it is not always the same individuals that are included in the «time after second dose » response groups. (B) SARS-CoV-2 vaccines used for patients with APS-1. Pfizer-BioNTech BNT162b2 mRNA vaccine (Pfizer), Oxford/AstraZeneca ChAdOx1-S vaccine (Astra Zeneca), Moderna mRNA-1273 vaccine (Moderna). (C) Number of vaccine doses for patients with APS-1 from Feb-2021 to Nov-2022. (D) Vaccine adverse events in patients with APS-1. Mild transient symptoms included low-grade fever, headache, and tiredness. A moderate symptom is here described as abnormal vaginal bleedings for several months after vaccination. (E) Patients with APS-1 (N = 32, black) and representative healthy controls (N = 2, red) spike IgG endpoint binding titers after SARS-CoV-2 vaccination. Threshold (dotted line) = 450. (F) IgG neutralization titers toward live human COVID-19/Norway/Bergen-01/2020 (Wuhan virus, GISAID accession ID EPI_ISL_541970) in patients with APS-1 (N = 29, black) after SARS-CoV-2 vaccination. Threshold (dotted red line) = 10. (E and F) Black arrow shows the first vaccine dose. Responses after vaccine doses 1, 2, and 3 are shown but time points for vaccine dose 2 and 3 vary and time points are therefore not shown in these figures.

Patients with APS-1 received no to three SARS-CoV-2 vaccinations, with 67% receiving only the Pfizer-BioNTech BNT162b2 mRNA vaccine (Figures 1A–1C). Two patients had both the BNT162b2 and Oxford/AstraZeneca ChAdOx1-S vaccines (6%), four patients were vaccinated with a combination of BNT162b2 and Moderna mRNA-1273 vaccines (12%), one patient (3%) was vaccinated with three doses of Moderna mRNA-1273, while one patient (3%) had one dose of each of the three different types of vaccine (Figures 1A and 1B). Three individuals did not know the vaccine types (9%) used. The mean interval between the first and second dose was 41 days (range 21–83 days) and the interval between the first and third dose was 230 days (range 82–382 days) (Table 1).

**Table 1:** The Norwegian APS-1 vaccine study

Included subjects	APS-1 patients	Matched healthy controls	Elderly persons
N	33	215	99
%Female	55	60	52
Mean age	46.0	44.5	86.0
Range age	19–76	23–82	65–99
Total samples	107 (range 1–6)	703 (range 3–4)	391 (range 3–4)
1^st^ vaccination	33	215	99
2^nd^ vaccination	33	213	99
3^rd^ vaccination	26	not included	not included
Samples before 1^st^ vaccine	33	0	0
Interval 1st to 2^nd^ dose (days)	41 (range 21–83)	21	21–22
Interval 1st to 3^rd^ dose (days)	230 (range 82–382)	not included	not included
Samples after/at 2^nd^vaccination (pre-3^rd^vaccination):			
Samples 3 weeks after 2^nd^ vaccine	12	213	99
Samples 6–8 weeks after 2^nd^ vaccine	12	211	97
Samples 5–6 months after 2^nd^ vaccine	15	304	99
Samples >9 months after 2^nd^ vaccine	27	75	96
Time points for vaccination 1–3 and sampling	Feb-Dec 2021	Feb-Nov 2021	Jan-Nov 2021

Sixteen patients reported mild vaccine-associated adverse events, including headache, low-grade fever, and tiredness (Figure 1D). We did not observe any specific pattern regarding adverse events related to type or timing of vaccines nor the number of vaccination doses received. One female patient who had three BNT162b2 doses experienced prolonged menstrual disorder with vaginal bleedings.

### Patients with APS-1 have functional, although lower, SARS-CoV-2 antibody vaccine responses compared to healthy adults

Serum SARS-CoV-2-specific (homologous Wuhan vaccine strain) antibody titers were measured by ELISA, Luminex, and virus neutralization assays pre, post, and in between three vaccine doses (Figure 1). All patients, except two, had detectable ELISA and neutralization antibodies, although one additional patient did not have neutralizing antibodies (Figures 1E, 1F, and S1). These results indicate that, in general, patients with APS-1 respond to vaccination by generating spike-specific antibodies. One patient on rituximab treatment still mounted an adequate vaccine response (Figure S1). Individual longitudinal IgG-measurements of spike-1/spike-2/RBD/nucleocapsid also demonstrated an adequate vaccine response, and that the third booster dose maintained high anti-spike-IgG levels (Figure S2). We additionally assayed 10 patients with APS-1 and two healthy controls at multiple time points (2–4) pre- and post-vaccination with monovalent vaccines against the original Wuhan strain (Table S2) for Omicron cross-reactive IgG antibodies. There were only three of the samples that had cross-reactive Omicron-neutralizing antibodies, two patients with APS-1 (one positive 47 days after third vaccination and one eight days after first vaccine) and one healthy control 112 days after third vaccination. In addition (∗ in Table S2), three patients were monitored after the initial study period and after confirmed infection by the SARS-2-CoV Omicron variant; two of these had higher Omicron-specific neutralizing antibodies while one remained negative.

We further measured autoantibodies against IFN-α2 and -ω before and after SARS-CoV-2 vaccination in 10 patients with APS-1, to investigate whether the interferon autoantibodies were affected by vaccination. No substantial pre-to-post variations were observed, supporting the fact that these APS-1 hallmark autoantibodies might not interfere with humoral SARS-CoV-2 vaccine responses (Figure S3).

**Figure 2:**
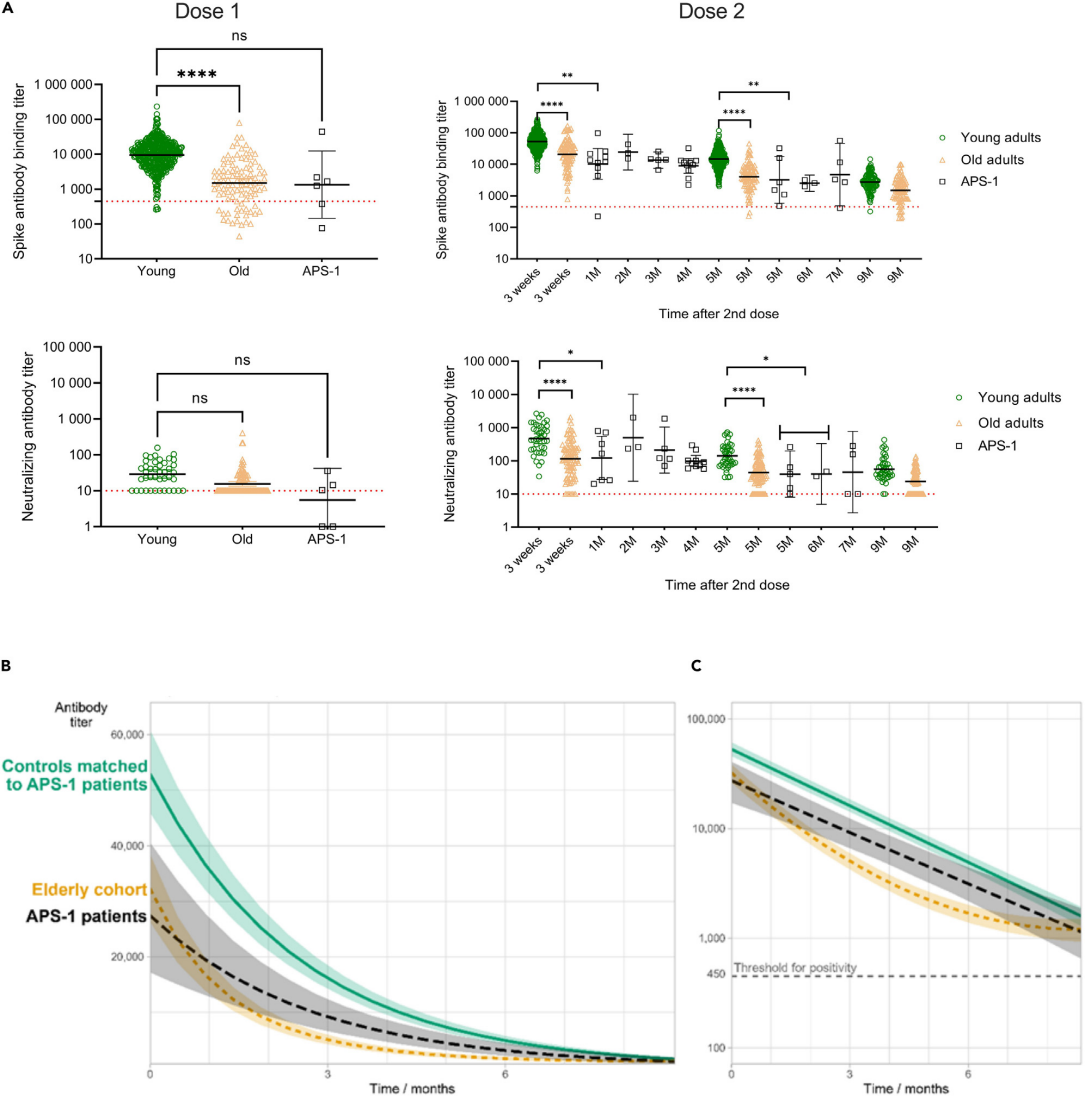
COVID-19 vaccine response in patients with APS-1 (A) Antibody responses in patients with APS-1, age- and gender-matched controls, and an older adult cohort after 1 and 2 vaccine doses, respectively. Only samples taken before the third vaccination have been included. Statistical differences between the three groups have been calculated when the time point for controls and patients with APS-1 matches (One-way ANOVA). (B and C) Model-based estimated IgG binding spike-specific vaccine responses for patients with APS-1, control cohort 1 (age- and gender-matched controls), and the older adult cohort. The figure shows the expected/median (log) response for a “typical” patient/control (i.e., a subject with the value zero for both random effects). The bands indicate 95% confidence intervals, calculated using bootstrapping (1,000 replications). The decay over time shows a linear slope for patients with APS-1 and matched controls, but follows a non-exponential pattern for elderly persons. Time 0 corresponds to 14 days after the second vaccine dose. (B) Linear axis for antibody titer. (C) Log-transformed axis, with the threshold for positivity indicated.

We next compared the vaccine responses of patients with APS-1 with age- and gender-matched healthy controls (control cohort I) and an elderly cohort (control cohort II) after their first and second vaccine doses. As seen in Figure 2A, the peak responses of APS-1 patients were lower than age-matched controls, and more similar to the elderly cohort, after both the first and second doses. This suggests a compromised vaccine response in patients with APS-1.

### Patients with APS-1 had senescent vaccine responses compared with healthy gender- and age-matched controls, but better than elderly persons

Models were generated to compare the kinetics of the vaccine responses in patients with APS-1 with matched healthy controls and elderly individuals (Figures 2B and 2C). Timing for the peak response was set as 14 days after first vaccine dose, representing time point “0” in the graphs. For the matched controls, the mean initial antibody titer for an average subject (where all random effects are zero) was 52592 (CI: 45448 to 60859). For patients with APS-1, this response was significantly reduced by 49% (1 – e^b^), to 26693 (b = −0.68, CI: −1.15 to −0.21, p < 0.001). For controls, the estimated growth constant c was −0.40 (CI: −0.42 to −0.37), corresponding to a half-life of 1.75 months (CI: 1.65 to 1.87), similar to patients with APS-1 (d = 0.04, CI: −0.05 to 0.13, p = 0.43). There was some inter-subject variation, measured by SDs of the random effects. For the response at t = 0, this variation was large, SD(A_i_) = 0.80. As ± 2 SDs represent most individuals, we expect initial IgG titers in controls to vary from about 10000 to 260000 (e^10.87 ± 2 × 0.80^) and to be 49% lower in patients with APS-1. The decay rate showed little inter-subject variation, SD(C_i_) = 0.09. This corresponds to half-lives varying between 1.2 and 3.2 months for both controls and patients with APS-1. Subjects with a high initial response had a shorter half-life, and a faster decay rate as the correlation between the two random effects was negative (−0.41, CI: −0.55 to −0.26). The elderly cohort had a more rapid initial decline and their decay curves were significantly different from the patients with APS-1 and matched healthy controls (p < 0.001 for all coefficients).

The predicted curves for all subjects show that the models fit the data (Figure S4). The small differences between the fitted and actual curves indicate that there is very little variation in the decay rates between subjects. However, we observed large differences in the initial vaccine response (at time 0, i.e. 14 days after first vaccine dose) between subjects. The panels were sorted by maximum estimated vaccine response at time 0; patients with APS-1 are mostly found at the bottom of the graph, indicating lower initial vaccine responses.

### SARS-CoV-2 infections in vaccinated patients with APS-1

**Figure 3:**
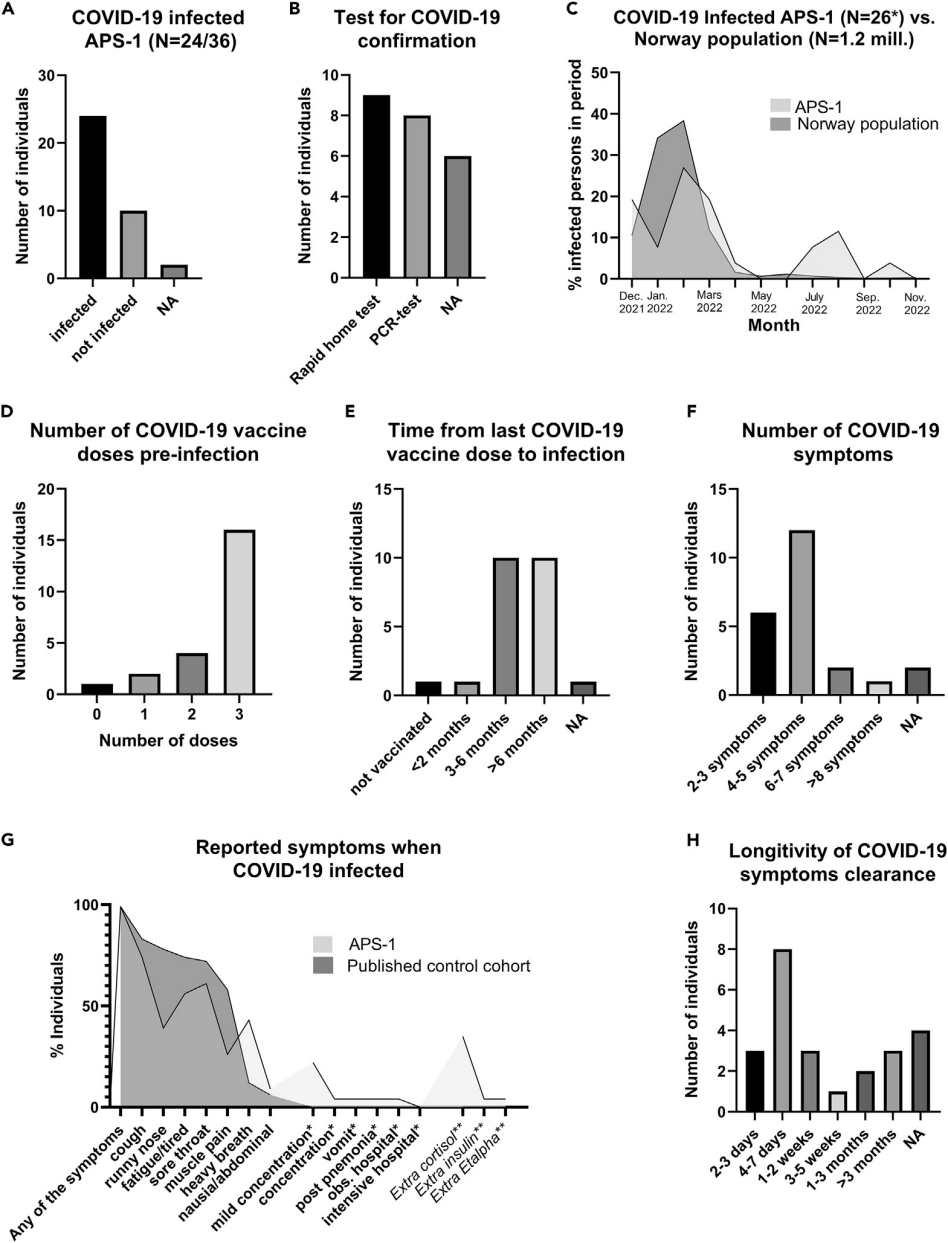
COVID-19 clinical study in Norwegian patients with APS-1 (A) Patients with APS-1 reporting SARS-CoV-2 infection. (B) Confirmation test that was used to determine SARS-CoV-2 infection. (C) Time point for SARS-CoV-2 infection in relation to publicly available numbers of infected persons in the Norwegian population from Dec-2021 to Nov-2022 [31, 32]. (D) Number of SARS-CoV-2 vaccine doses before infection. (E) Time from last COVID vaccine dose to infection. (F) Number of symptoms after SARS-CoV-2 infection. (G) Nature of the COVID-19 symptoms in patients with APS-1 and a published control cohort [25]. Only symptoms that were reported in both studies and only for patients with APS-1 (∗) are shown. Note that we did not ask our patients regarding taste/smell disturbances, appetite, headache, and sneezing and the reference paper did not describe concentration issues nor had data on hospitalization. Extra use of medications when infected is indicated by ∗∗. (H) Time to full reconstitution after SARS-CoV-2 infection.

Twenty-four patients with APS-1 (66%) were infected by SARS-CoV-2 virus during the study period based on symptoms and a positive PCR or rapid antigen test (lateral flow test); all of these except one child were infected post-vaccination (Figures 3A and 3B). We confirmed that 19 of the patients with APS-1 had not been infected prior to vaccination with any measurable anti-nucleocapsid antibodies (Figure S2). Infections occurred in the period December 2021–November 2022, and two patients had two confirmed SARS-CoV-2 infections. The timing of infections matched the epidemiology of reported COVID-19 cases in Norway during the Omicron wave (Figure 3C), when >90% of the infected Norwegian cases were caused by the Omicron variant.^23^^,^^24^ Most of the patients had received three doses of vaccine at the time of infection (Figures 3D and 3E).

Patients with APS-1 reported between two and ten symptoms after SARS-CoV-2 infection, with a median of four (Figures 3F and 3G). The COVID-19 symptoms were usually mild, including fever, muscle pain, fatigue, runny nose, cough, sore throat, and concentration problems, resembling a Norwegian cohort of adult vaccinated patients infected with Omicron.^25^ Most patients had symptoms of COVID-19 for less than a week similarly to previous observations in healthy people of two to eight days,^25^ although five subjects had persisting cough and/or fatigue for longer durations (Figure 3H). One patient with APS-1 had two episodes with pneumonia about six months after COVID-19. Another patient complained about dyspnea six months after the infection, but later improved. One patient was hospitalized for observation while SARS-CoV-2 infected, but had no severe outcome.

During the study period, two patients died of causes unrelated to COVID-19. Eleven patients reported taking extra medications when infected; Nine with adrenal insufficiency took glucocorticoids, one with type 1 diabetes increased insulin doses, while one had hypertension and also administrated more of the active vitamin D-drug Etalpha (Figure 1G).

## Discussion

In the present study, we show that COVID-vaccinated patients with APS-1 elicit antibody responses with adequate increase in SARS-CoV-2 Wuhan-specific spike IgG and neutralizing antibodies, and with sufficient levels to protect them from severe COVID-19. However, the patients with APS-1 show a senescent humoral response after vaccination similarly to elderly individuals, although probably with different underlying biological mechanisms. SARS-CoV-2 vaccination of young individuals with APS-1 has recently been shown to confer an adequate viral-specific humoral immune response.^22^ Here, we confirm these findings in adult patients with APS-1. However, even though patients with APS-1 appear to have similar longitudinal decline in antibody titer as healthy controls, the lower peak response results in premature loss of functional antibodies. Hence, these patients need to be boosted with earlier and more frequent vaccine doses than healthy young individuals. As none of the patients with APS-1 included in this study reported severe adverse events after vaccination, we support previous recommendations regarding acceptable safety and tolerability of SARS-CoV-2 vaccines in immune-compromised individuals.^26^^,^^27^^,^^28^^,^^29^ Notably, only two out of 10 randomly assayed patients with APS-1 and one of two healthy controls elicited low Omicron-specific neutralization antibody responses after vaccination with monovalent SARS-CoV-2 vaccines. However, two out of three patients with APS-1 sampled post Omicron infection had Omicron-neutralizing antibodies. This shows that the cross-reactive humoral Omicron response after monovalent Wuhan vaccination is modest, which has also been previously shown for healthy controls and different patient groups, explaining the breakthrough infection rates.^30^^,^^31^^,^^32^^,^^33^^,^^34^^,^^35^

In the present cross-sectional design including 95% of the living Norwegian APS-1 population with 24 infected SARS-CoV-2 cases, none experienced severe illness. Only two patients complained about tiredness and dyspnea months after the infection, which might indicate delayed clearance of SARS-CoV-2 virus, but equally likely represent common post-COVID-19 symptoms. Our observations contrast with previous studies of severe COVID-19 in unvaccinated patients with APS-1,^5^^,^^6^^,^^8^^,^^15^^,^^36^ although the infecting variant may have played a role. Most of our patients were infected by Omicron, which was the leading cause of COVID-19 in Norway from January 2022.^23^ Alpha and Delta variants, which likely were the dominating variants in the previous studies, are less infective than Omicron, but can confer more severe disease.^37^^,^^38^^,^^39^ Although mRNA vaccination offers some protection against Omicron infections (21%–44% depending on the outcome measures and time since vaccination), booster vaccinations do improve effectiveness despite variants which are able to escape vaccine-induced immunity.^40^ Nevertheless, our data show that vaccination protected the patients with APS-1 against severe illness during the Omicron period.

The role of IFN-Is and autoantibodies against IFN-Is in relation to COVID-19 severity has been debated after it was demonstrated that up to 10% of hospitalized SARS-CoV-2-infected patients had circulating IFN-I antibodies.^5^^,^^6^^,^^7^^,^^8^^,^^9^^,^^10^^,^^15^^,^^21^ Patients with APS-I have a constant high level of neutralizing IFN-I autoantibodies that are associated with lower expression of IFN-regulated genes in isolated peripheral blood mononuclear cells, plasmacytoid dendritic cells, monocytes, and monocyte-derived dendritic cells.^41^ An example of the reverse situation is individuals with Down syndrome, who have initially increased expression of the receptors for IFN-Is (IFNAR1/2) encoded from chromosome 21. These individuals show higher levels of IFN-I transcripts at first, which by feedback lead to lower subsequent IFN-I responses.^42^ This protects individuals with Down syndrome from SARS-CoV-2 infections but may lead to severe disease if infection does occur. We can only speculate if the IFN-autoantibodies in APS-1 (or in other IEI or elderly people) are a protective mechanism aimed at protecting internal organs from IFN-I-related tissue damage. This is underpinned by the presence of immunoreactive IFN-α in beta cells of diabetic patients and the few patients with APS-1 without IFN-α autoantibodies, which are more prone to type 1 diabetes and thyroid disease.^42^^,^^43^ Such mechanisms could then be partly beneficial during COVID-19 illness but devastating for local lung tissue if the patient does not have protective immunity from vaccination or previous infection to promote SARS-CoV-2 clearance. Indeed, IFNs in general play a regulatory role in adaptive immunity, by “Th1 skewing,”^44^ and local variations of different IFN-I subtypes may also impact viral host defense in patients with APS-1 as IFN-β and IFN-γ are usually not targeted by the autoantibodies.^45^ Interestingly, Hetemaki and colleagues recently showed that high levels of IFN-α4 autoantibodies in patients with APS-1 are a risk factor for severe herpesvirus infections.^46^ Notably also, it has been reported that IFN-λ1, being an IFN-III cytokine which is sometimes targeted by APS-1 antibodies,^47^ plays a protective role in SARS-CoV-2 infections. Recently, the Omicron variant was found to induce IFN-λ1, while other earlier variants did not, suggesting that IFN- λ1 provides antiviral protection against Omicron in the upper respiratory tract, thus preventing spread to lower lung tissue.^48^ The different functionalities and response strengths of the IFN-subtypes may hence influence the severity of SARS-CoV-2 infections. Also chemokine autoantibodies measured post-SARS-CoV-2 infection has been found to correlate with severity of COVID-19, adding to the complexity of how cytokines/chemokines interfere with host immunity toward viruses.^49^ How then this plays out in patients with APS-1 with the myriad of immunoregulatory cells, cytokines, hormones, signaling molecules, and autoinflammation is yet to be determined. Notably, the IFN-I autoantibody level did not vary after the first, second, or third vaccination in our APS-1 cohort, suggesting that vaccination does not affect autoantibody responses. How this may impact upon SARS-CoV-2 virus infections is still unresolved.

In conclusion, we have shown that COVID vaccination of patients with APS-1 is safe and protects from severe outcomes of subsequent SARS-CoV-2 infections. This demonstrates that continuous presence of IFN-I autoantibodies are not major determinants for manifestations and severity of COVID-19 in these patients.

### Limitations of the study

Sampling of patients who live across Norway is difficult to standardize regarding timing after vaccination, and patients were immunized with different vaccines subjected to vaccine availability. This is also the reason why T cell response studies were not possible. Our models are all based on relatively restricted numbers of observations. For example, we did not have enough samples per subject (and variation in the sample time points) to differentiate our ad-hoc model for the elderly cohort with a squared term from other models with a similar number of parameters but other decay curve shapes, so we do not report the coefficient estimates for this cohort. The relatively mild phenotypes of Norwegian patients compared to patients from Russia and USA [14, 18, 41, 42] could reduce the generalization of our findings, although genotypically Norwegian and USA patients are similar.

## STAR★Methods

### Key resources table

**Table:** 

REAGENT or RESOURCE	SOURCE	IDENTIFIER
**Antibodies**		

anti-SARS-CoV-2 nucleoprotein rabbit monoclonal IgG	Sino Biological	40143-R001; RRID: AB_2827974
anti-rabbit biotinylated goat IgG (H + L)	Southern Biotech	BA-1000-15
goat anti-human IgG–horseradish peroxidase (HRP) conjugated secondary antibody	Sigma	A0170; RRID: AB_257868

**Bacterial and virus strains**		

Live virus D614G human COVID-19/Norway/Bergen∗01/2020 (Wuhan)	Cox Laboratory Trieu et al.^50^	GISAID accession ID ISL_541970
Live virus SARS-CoV-2/human/NOR/Bergen-05/2022 (Omicron);	Cox Laboratory	NCBI accession number GenBank: ON222956
Modified spike from SARS-CoV-2 used in ELISA	Krammer Laboratory Amanat et al.^51^ Also BEI Resources (https://www.beiresources.org/).	NCBI, GenBank IDs MT380724.1 and MT380725.1
Encephalomyocarditis virus	Meager Laboratory Meager et al.^12^	

**Biological samples**		

Sera from APS-I patients	Registry and biobank for organ specific autoimmune disorders, Husebye Laboratory, Norway	
Sera from healthy controls (control group I; n = 215, 60% females, mean age 44.5 years)	Cox Laboratory Trieu et al.; Hansen et al.^50^^,^^52^	
Sera from healthy older adults (control group II, n = 99, 52% females, mean age 86.0 years) Hansen et al.^52^	Cox Laboratory Trieu et al.; Hansen et al.^50^^,^^52^	
Controls for ELISA. A prepandemic sera pool, a hospitalized patient serum, and the human monoclonal antibody reactive to both SARS-CoV-1 and 2 (CR3022) were used as controls	Cox Laboratory Trieu et al.; Hansen et al.^50^^,^^52^	
Two healthy controls (one male 38 years of age and one female 45 years of age), controls for Luminex assay and omicron neutralization assay	Husebye Laboratory	

**Chemicals, peptides, and recombinant proteins**		

pcDNA3-vector	Invitrogen Addgene: Vector Database - pcDNA3	A kind gift from Per Knappskog, Helse-Bergen, Norway
pcDNA3.1-TOPO-vector	Invitrogen	K490001
IFN-α2a protein	Hoffmann-La Roche, Basle, Switzerland, Meager et al.^12^	
IFN-α8 protein	Glaxo-Wellcome, Beckenham, Kent, UK, Meager et al.^12^	
IFN-ω protein	Bender & Co., Vienna, Austria, Meager et al.^12^	
Substrate *o*-phenylenediamine dihydrochloride	Sigma-Aldrich	P6662
SIGMAFAST OPD, *o*-phenylenediamine dihydrochloride	Sigma-Aldrich	P9187

**Critical commercial assays**		

Cell free protein expression; *In vitro* transcription and translation	Promega, Madison, Wisconsin, US	P1420, P1430, P1440
BioPlex Pro Human IgG SARS CoV Serology assay	BioRad, Hercules, California, US	12014634

**Experimental models: Cell lines**		

Human glioblastoma cell line 2D9	Provided from Dr W. Däubener Däubener et al.^53^	
Vero cells	Cox Laboratory Trieu et al.^50^	

**Recombinant DNA**		

Human IFN-α2 cDNA	Invitrogen, Carlsbad, CA, USA; for cloning protocol see Hapnes et al.^54^	
Human IFN-ω cDNA	Vical Inc. San Diego, CA, USA; for cloning protocol see Oftedal et al.^55^	
IL-22-cDNA-molecules	OriGene, Rockville, MD, USA; for cloning protocol see Oftedal et al.^56^	
Human IFN-α8 cDNA	For cloning protocol see Hapnes et al.^54^	
21-hydroxylase, 17-hydroxylase, glutamic acid decarboxylase-65, NACHT leucine-rich-repeat protein 5, aromatic-L amino acid decarboxylase, tryptophan hydroxylase, tyrosine hydroxylase, and side-chain cleavage enzyme.	Results adopted from Bruserud et al.^16^	

**Software and algorithms**		

GraphPad Prism 9.3.1	Dotmatics, Prism - GraphPad	
R packages ‘nlme’ version 3.1–161	José Pinheiro et al.^57^	
R version 4.2.1	R Core Team^58^	

**Other**		

ELISA was used to determine the Spike-specific endpoint IgG antibodies in sera from patients and controls following the protocol described previously Trieu et al.^50^ A detailed protocol for protein expression and ELISA setup for Spike-specific IgG measurements	https://currentprotocols.onlinelibrary.wiley.com/doi/full/10.1002/cpmc.100 Reference; Trieu et al.^50^	
Virotrol SARS CoV-2 single level control	BiRad, Hercules, California, US	200300A
Filter plates for RIA	Multiscreen Filter Plates, BV 1.3 μm;	MABVN0B50, Millipore

### Resource availability

#### Lead contact

Further information and requests for resources and reagents should be directed to and will be fulfilled by the lead contact, Anette S. Bøe Wolff (anette.boe@uib.no).

#### Materials availability

• There are restrictions on the availability of the live virus Wuhan D614G human COVID-19/Norway/Bergen∗01/2020 (GISAID accession ID ISL_541970) and the SARS-CoV-2/human/NOR/Bergen-05/2022 (Omicron-variant); NCBI accession number GenBank: ON222956 due to shipping restrictions of BSL-3 pathogens.
• Plasmids with cDNA-inserts for use in radioimmunoassay and reagents for in house neutralization assay of interferons have been generated in previous studies (referred to in the methodology section) and can be available upon request and written formal MTA documents.

### Experimental model and study participant details

#### Patients

All APS-1 patients are included in the Norwegian registry for organ-specific autoimmune disorders and have been previously described (Table S1). Diagnosis was confirmed by clinical criteria for this syndrome, *AIRE* mutational analysis, and/or autoantibody screening against IFN-ω.^16^ The study did not interfere with patient treatment, and patients used routine medication, such as glucocorticoids to restore physiological cortisol levels for those with adrenal insufficiency. One patient was receiving immune suppressive treatment (Rituximab).

Thirty-six APS-1 patients (n = 36; 34 adults, 2 children) (Table S1) were included in the COVID-19 course study which constitutes a short questionnaire regarding SARS-CoV-2 vaccination and infection. The questionnaire concerned vaccination regime, timing, adverse events, confirmed SARS-CoV-2 infection (reverse transcriptase polymerase chain reaction (RT-PCR) or lateral flow test), and if relevant; symptoms and COVID-19 disease course. The patients were also asked about medication taken during the time of this study.

APS-1 patients provided serum samples before, between, and after COVID-19 vaccinations. Thirty-three adult patients (55% females, mean age 46.0 years) provided samples and were followed after the first vaccination in February 2021 until November 2022 (Tables 1 and S1). Patients received various combinations with different time intervals of the three vaccine types Pfizer-BioNTech BNT162b2 (mRNA type), Oxford/AstraZeneca (ChAdOx1-S) (protein in adenoviral vector) and Moderna mRNA-1273. Most patients received the BNT162b2 (Pfizer) vaccine (67%) (Table 1, Figure 1B).

#### Healthy controls

Age and gender-matched controls for the APS-1 patients were health care workers (control group I; n = 215, 60% females, mean age 44.5 years)^50^ and healthy older adults (control group II, n = 99, 52% females, mean age 86.0 years)^52^ followed pre- and post- SARS-CoV-2 vaccination (Table 1). The control subjects were all vaccinated with two doses of BNT162b2 (Pfizer) with an interval of three weeks (21–24 days) between the first and second dose (Table 1). None of the healthy controls received a third vaccine dose.

#### Ethics

All subjects provided written informed consent before inclusion in the study. Studies on APS-1 patients within the Norwegian registry for organ specific autoimmune disorders were approved by the Regional Ethical Committee (REC) of Norway (2009/2555, 2018/1417, and 285891). The age-and gender-matched healthy controls (n = 215) and elderly (n = 99) were used as comparator groups (REC: 118664 and 218629) and are registered in NIH clinical trials.gov (NCT04706390).

#### Sources of SARS-CoV-2

Neutralization assays for SARS-CoV-2 antibodies were conducted on live virus D614G human COVID-19/Norway/Bergen∗01/2020 (GISAID accession ID ISL_541970) and SARS-CoV-2/human/NOR/Bergen-05/2022 (omicron); NCBI accession number GenBank: ON222956. These viruses were isolated in house from RT-PCR–confirmed Norwegian patients and propagated in Vero cells before use.^50^

Constructs used for protein expression of spike from SARS-CoV-2 in cell systems used in ELISA were obtained from the Krammer Laboratory^51^ (NCBI, GenBank IDs MT380724.1 and MT380725.1 and BEI Resources (https://www.beiresources.org/).

#### Sources of cDNAs for radiolabeled cell free protein expression

Human IFN-α2 cDNA in the pENTR221 vector was TA-cloned into PCR2-TOPO following the manufacturer’s protocol (Invitrogen, Carlsbad, CA, USA) and then subcloned into the recipient vector pcDNA3. Human IFN-ω cDNA was subcloned from the VR1055 cloning vector (Vical Inc. San Diego, CA, USA) into pcDNA3, and two purchased IL-22-cDNA-molecules (OriGene, Rockville, MD, USA) were subcloned in tandem into pcDNA3. Human IFN-α8 cDNA in pENTR221 was first subcloned into pDEST14 using Clonase (Invitrogen) followed by TA subcloning into the pcDNA3.1-TOPO-vector (Invitrogen).^54^^,^^56^^,^^59^

#### Sources for interferon AVINA

The human glioblastoma cell line 2D9 was provided from Dr W. Däubener.^53^

#### Sources of cytokines for ELISA

IFN-α2a was from Hoffmann-La Roche, Basle, Switzerland and IFN-α8 was from Glaxo-Wellcome, Beckenham, Kent, UK. IFN-ω was from Bender & Co., Vienna, Austria.

### Method details

#### Autoantibodies in APS-1 patients

Binding and neutralization autoantibodies in APS-1 patients against organ-specific targets, IFN-α2, IFN-α8, IFN-ω and IL-22 were analyzed by radioimmune assay (RIA)^54^^,^^56^^,^^59^ or enzyme linked immunosorbent assay (ELISA)^12^ and antiviral interferon neutralization assay (AVINA).^12^

• RIA for binding autoantibodies

Plasmids with interferon/interleukin cDNAs were used as templates in cell free protein expression experiments (*in vitro* transcription and translation, Promega, Madison, Wisconsin, US) to produce ^35^S-labeled proteins.

For radioimmunoassay (RIA) in microtiter 96-well format, radiolabeled proteins (30000–50000 cpm per well) were mixed with serum (5 μL) in triplicates and incubated at 4°C over night. On day two, this antigen:anibody mixture was incubated with washed Protein-A-Sepharose, which will bind to the Fc-part of IgG-molecules. When applying these mixtures on filter plates (MABVN0B50, Millipore)), followed by 45 min incubation, washing and drying of the filters, levels of antbody binding can be estimated based on the radioactive signal from the bound protein to antibody, held up on the filter by the Sepharose beads. The RIA-buffer included 0.1% dithiothreitol for cytokine antibodies to prevent cross-linking and aggregation during incubation, and hence provide a more efficient immunoprecipitation. Results are expressed as binding indices ((cpm sample − cpm negative control)/(cpm positive control − cpm negative control) × 1,000). The negative control was serum pooled from healthy blood donors, and the positive controls were from APS I patients with medium/high autoAb levels. The threshold for positivity was set as the mean of the indices of 80–150 healthy blood donors, plus three standard deviations. The methods have been described in detail previously.^12^^,^^54^^,^^55^^,^^60^ The organ-specific targets for autoantibody radioimmune analysis included in Table S1 were 21-hydroxylase, 17-hydroxylase, glutamic acid decarboxylase-65, NACHT leucine-rich-repeat protein 5, aromatic-L amino acid decarboxylase, tryptophan hydroxylase, tyrosine hydroxylase, and side-chain cleavage enzyme, and these have all been reported for the APS-I patients previously.^16^

• ELISA for binding cytokine autoantibodies

Round-bottomed microtitre wells (Dynatech) were coated with 100 μL per well IFN at 2 μg protein/mL concentration in PBS for 2 h at room temperature. Wells were then blocked with 1% human serum albumin in PBS overnight at 4°C. Patient sera were diluted serially and added to coated wells, incubated for 2 h at room temperature, removed and wells washed with 0 · 1% Synperonic solution. Antihuman IgG-peroxidase conjugate (Sigma Chemical Co. Ltd), 0 · 1 mL/well of 1:1000 dilution was added to all wells and incubation continued at room temperature for a further 2 h. After washing, orthophenylene diamine substrate solution was added and color development terminated after 30 min by addition of 2 M H2 SO4 (0 · 05 mL/well). Absorbance was read at 490 nm.

• AVINA for neutralization antibodies

The human glioblastoma cell line 2D9 was pre-treated with diluted cytokine preparations preincubated for 1 h with serial dilutions of test sera. The cells were then added to encephalomyocarditis virus for 24 h. The cells were subsequently stained with 0 · 05% amido blue-black and fixed with 4% formaldehyde solution in acetic acid buffer, destained with 0 · 15 mL of 0 · 05 m NaOH solution and absorbances was read at 620 nm. The neutralizing antibody titer was calculated as the dilution of serum that reduces 10 LU/mL of IFN to 1 LU/mL (cut-off for positivity was 100).^12^

#### SARS-CoV-2 spike-specific IgG ELISA

ELISA was used to determine the Spike-specific endpoint IgG antibodies in sera from patients and controls following the protocol described previously.^50^ A detailed protocol for protein expression and ELISA setup is available (https://currentprotocols.onlinelibrary.wiley.com/doi/full/10.1002/cpmc.100) with in house optimization and modifications described in Trieu et al. specifically using Nunc maxisorp ELISA plates, anti-human IgG HRP (Sigma) and the substrate 3,3′,5,5′-tetramethylbenzidine (TMB; BDbiosciences). Sera from patients and controls were titrated in triplicates, starting from 1:100, to detect IgG binding to the spike proteins. A prepandemic sera pool, a hospitalized patient serum, and the human monoclonal antibody reactive to both SARS-CoV-1 and 2 (CR3022) were used as controls. Endpoint titers were calculated as the reciprocal of the sample dilution giving an absorbance value of three SD above historical pre-pandemic sera, N = 128 (Threshold: 450). For this assay, two healthy controls who had also been sampled before (one male 38 years of age and one female 45 years of age), between and after the vaccine doses were included in the same assay as references (also used as controls for the Luminex-assays).

#### Neutralization assay for SARS-CoV-2

We performed a microneutralization assay with live Wuhan virus D614G human COVID-19/Norway/Bergen∗01/2020 (GISAID accession ID ISL_541970) in a Biosafety Level 3 Laboratory as described by Trieu et al.^50^ The microneutralization assay was also replicated in 26 samples from 10 APS-1 patients using the SARS-CoV-2/human/NOR/Bergen-05/2022 (omicron) variant; NCBI accession number GenBank: ON222956. Sera were serially diluted (minimum 1:10) and mixed with 100 tissue culture infectious dose 50% (TCID50) virus in 96-well plates. This was incubated for 1 h at 37°C before transferring to 96-well plates pre-seeded with Vero cells for 24-h. Cells were permeabilized and fixed with methanol and 0.6% H2O2 and incubated with the anti-SARS-CoV-2 nucleoprotein rabbit monoclonal IgG (Sino Biological), followed by anti-rabbit biotinylated goat IgG (H + L) (Southern Biotech), extravidin-peroxidase (Sigma-Aldrich) and substrate *o*-phenylenediamine dihydrochloride (Sigma-Aldrich). The absorbance was read at 450 nm. Neutralization titers were calculated as the reciprocal of the serum dilution which gave 50% inhibition of virus activity. Values below the negative threshold (10) were calculated as 1.

#### Semi-quantification of SARS-CoV-2 antibodies

Semiquantitative measurements of levels of antibodies against four different SARS-CoV-2 proteins (Nucleocapsid/“the receptor binding domain” (RBD)/Spike1/Spike2) in patient sera were conducted using Luminex technology and the BioPlex Pro Human IgG SARS CoV Serology assay (BioRad #12014634) on a BioPlex200 (BioRad) with the BioManager Software 6.1 (BioRad) according to the manufacturer’s specifications. For quantification, the Virotrol SARS CoV-2 single level control (Bio-Rad #200300A) was used to create a standard curve. Nineteen adult APS-1 patients with longitudinal samples were assayed and two healthy controls (see ELISA) were included as references. Previous SARS CoV-2 infections were confirmed by the presence of antibodies to the nucleocapsid protein.

### Quantification and statistical analysis

#### Figures and models

• Software

GraphPad Prism 9.3.1 was used to make figures for the Luminex and ELISA results. Models were fitted using the R packages ‘nlme’ version 3.1–161^57^ and ‘lme4’ version 1.1.31^61^ on R version 4.2.1^58^ (see below).

• *Model for SARS-CoV-2-antibody endpoint titers*

With the aim to generate a model for the longitudinal spike-IgG-response (ELISA-based), we first performed a basic visualization of the data (not shown). We found that APS-1 patients and matched healthy controls (control cohort I) followed the same pattern and could be modeled with an exponential decay curve, whereas the elderly cohort (control cohort II) had different kinetics. For the APS-1 patient and healthy controls, we modeled an exponential reduction curve with a different decay rate and initial response for each individual. To this end, we fitted a linear mixed-effects model on the log-responses. For each individual *i* at time *t*, the log-response was modeled as

**Table:** 

log(response_*it*_) =	*a* + *A*_*i*_ + *b* × group_*i*_	Log-response at *t* = 0
	+ (*c* + *C*_*i*_ + *d* × group_*i*_) × *t*	Growth over time
	+ *ε*,	Residual error

where *t* is measured in months (= 30 days), with *t* = 0 corresponding to 14 days after the second dose, as we anticipated this as the peak endpoint IgG titers. The variable group was set to 1 for APS-1 patients and 0 for controls. The parameter *a* measures the initial log-response for a ‘typical’ control (i.e., the mean log-response at *t* = 0 for a control with both random effects equal to zero), and *b* (expected to be negative) the ‘extra’ initial log-response for an APS-1 patient. The parameter *c* is the ‘growth constant’ (negative for decay) for a ‘typical’ *control* (with half-life = −log(2)/c), and *c* + *d* the growth constant for a ‘typical’ *APS-1 patient*. *A*_*i*_ and *C*_*i*_ are zero-mean normal variables, allowed to correlate, and measuring the differences between individual *i* and a ‘typical’ subject in initial response and decay rate, respectively. The residual *ε* is an independent zero-mean normal variable.

The model provided an acceptable fit for the patients and matched healthy controls, as measured by diagnostic plots of residuals and estimated random effects. To optimize fitness to the model for the *elderly* control, we made an ad-hoc adaption by adding a ‘squared time’ term for this cohort, in addition to separate terms for the initial log-response and decay. This resulted in a model with acceptable fit for all three groups.

Only measurements from 14 days after the second vaccine dose up to the third dose were included. Data from subjects with clearly non-monotone decay curves were excluded. Common reasons for the discrepant/non-monotone curves were: 1) third dose received, but not recorded, 2) SARS-CoV-2 infection, 3) laboratory/measurement errors. In all, data for 25 patients, 136 healthy controls, and 86 elderly controls were included (247 individuals, 576 measurements) in the model. The number of samples per subject was three (47%), two (39%), or one (14%). Patients with only one sample were included in the model, as they do provide valuable information on the mean log-response at *t* = 0.

## Data Availability

• The cytokine autoantibody and SARS-CoV-2 antibody data reported in this study cannot be deposited in a public repository because these are patient/personal data, belonging to a medical registry (for patients), the elderly and healthy controls who were health care workers. These individuals have signed informed consent forms for participation in research which we must adhere to. To request access to parts of de-identifiable data, contact Registry for organ specific autoimmune disorders (ROAS), Haukeland University hospital, Norway, e-mail: Eystein.husebye@helse-bergen.no or for elderly and healthy controls (Rebecca Cox, Rebecca.jane.cox@uib.no). • This paper does not report original code. • Any additional information required to reanalyze the data reported in this paper is available from the lead contact upon request. Additional resources• The age-and gender-matched healthy controls and elderly who were used as comparator groups were registered in NIH clinical trials.gov (NCT04706390).
