# Supplementary material for: Vaccination prevents severe COVID-19 outcome in patients with neutralizing type 1 interferon autoantibodies
Source: iScience. 2023 Jun 9;26(7):107084. doi: 10.1016/j.isci.2023.107084 (PMC10251722; doi:10.1016/j.isci.2023.107084)
Supplement: Document S1. Figures S1–S4 and Tables S1 and S2 [file mmc1.pdf]

iScience, Volume *26*

## Supplemental information

# Vaccination prevents severe COVID-19 outcome in patients with neutralizing type 1 interferon autoantibodies

**Anette S.B. Wolff, Lena Hansen, Marianne Aa. Grytaas, Bergithe E. Oftedal, Lars Breivik, Fan Zhou, Karl Ove Hufthammer, Thea Sjøgren, Jan Stefan Olofsson, Mai Chi Trieu, Anthony Meager, Anders P. Jørgensen, Kari Lima, Kristin Greve-Isdahl Mohn, Nina Langeland, Rebecca Jane Cox, and Eystein S. Husebye**

**Supplementary material overview:**

Supplementary Table S1: Included APS-1 patients and autoantibodies in the SARS-CoV-2/COVID-19-studies

Supplementary Table S2: Neutralization antibody response against the SARS-CoV-2 omicron variant in APS-1 patients

Supplementary Figure S1: Neutralizing and binding spike IgG antibodies in three APS-I patients with low vaccine responses. Response for one patient on Rituximab.

Supplementary Figure S2: Antibody titers of longitudinal sampling of 19 APS-I patients and two healthy controls measured by a 4-plex Luminex kit.

Supplementary Figure S3: Autoantibodies against IFN-Is in patients with APS-1 pre- and post-vaccination (N=10) with SARS-CoV-2 and after infection.

Supplementary Figure S4: Measured spike IgG binding vaccine responses along with model-based fitted decay curves for each subject.

**Table S1: Included APS-1 patients and autoantibodies in the SARS-CoV-2/COVID-19-studies**

| | Patient number | 1 | 2 | 3 | 4 | 5 | 6 | 7 | 8 | 9 | 10 | 11 | 12 | 13 | 14 | 15 | 16 | 17 | 18 | 19 | 20 | 21 | 22 | 23 | 24 | 25 | 26 | 27 | 28 | 29 | 30 | 31 | 32 | 33 | 34 | 35 | 36 | 37 | 38 |
|---|---|---|---|---|---|---|---|---|---|---|---|---|---|---|---|---|---|---|---|---|---|---|---|---|---|---|---|---|---|---|---|---|---|---|---|---|---|---|---|
| Demographics: | Gender | | | | | | | | | | | | | | | | | | | | | | | | | | | | | | | | | | | | | | |
| | Age group | | | | | | | | | | | | | | | | | | | | | | | | | | | | | | | | | | | | | | |
| Medications: | Corticoids for systemic use | | | | | | | | | | | | | | | | | | | | | | | | | | | | | | | | | | | | | | |
| | Vitamins and minerals (Vit D, Mg, Ca) | | | | | | | | | | | | | | | | | | | | | | | | | | | | | | | | | | | | | | |
| | Heart-related | | | | | | | | | | | | | | | | | | | | | | | | | | | | | | | | | | | | | | |
| | Digestion, systemic hormones, metabolism | | | | | | | | | | | | | | | | | | | | | | | | | | | | | | | | | | | | | | |
| | Other medications | | | | | | | | | | | | | | | | | | | | | | | | | | | | | | | | | | | | | | |
| Autoantibodies | IFN-ω Binding/Neutr. Abs | | | | | | | | | | | | | | | | | | | | | | | | | | | | | | | | | | | | | | |
| | IFN-α Binding/Neutr. Abs | | | | | | | | | | | | | | | | | | | | | | | | | | | | | | | | | | | | | | |
| | Binding IFN-α8 Abs | | | | | | | | | | | | | | | | | | | | | | | | | | | | | | | | | | | | | | |
| | IL22 Binding/Neut. Abs | | | | | | | | | | | | | | | | | | | | | | | | | | | | | | | | | | | | | | |
| | Organ specific Abs N=8 | | | | | | | | | | | | | | | | | | | | | | | | | | | | | | | | | | | | | | |
| Included in substudy: | Vaccine study: ELISA N=32 | | | | | | | | | | | | | | | | | | | | | | | | | | | | | | | | | | | | | | |
| | Vaccine study: Neutralization N=29 | | | | | | | | | | | | | | | | | | | | | | | | | | | | | | | | | | | | | | |
| | Vaccine study: Luminex 4-plex N=19 | | | | | | | | | | | | | | | | | | | | | | | | | | | | | | | | | | | | | | |
| | Questionnaire N=36 | | | | | | | | | | | | | | | | | | | | | | | | | | | | | | | | | | | | | | |

***Gender:*** *Female (orange); Male (blue).*

***Age group:*** *0. 8-17 years (white); Then gradually darker according to age raise: 1. 18-32 years (very light grey); 2. 33-42 years (light grey); 3. 43-52 years (grey); 4. 53-62 years (dark grey); 5. 63-77 years (very dark grey).*

***Medication (self-reported):*** *Positive: white; Not known: checkered; Negative: black. Other medications include: anemia, antifungals, antihistamins, nervrologica, respirational treatment, sex hormones and more.*

***Autoantibodies in serum:*** *Positive: white; Not analyzed: checkered; Negative: black. >1 organ specific autoantibody type: Black. Both binding and neutralizing antibodies have been analyzed. If at least one of them shows positivity, it is denoted as positive here.*

***Included in sub-study:*** *If included: white. If not included: black. For number 34-38: no samples were available for the vaccine study*

**Table S2: Neutralization antibody response against the SARS-CoV-2 Omicron variant in APS-1 patients**

| ID | post 1st vacc. (d) | post 2d vacc. (d) | post 3rd vacc. (d) | number of vaccines | other info | infected with omicron pre-sampling? | BA.1 MN titer |
|---|---|---|---|---|---|---|---|
| Pt 2 | before vaccine | Na | Na | 0 | low Wuhan-variant response | No | ND |
| Pt 2 | 210 | 189 | Na | 2 | low Wuhan-variant response | No | ND |
| Pt 4 | 2 | Na | Na | 1 | | No | ND |
| Pt 4 | 189 | 166 | Na | 2 | | No | ND |
| Pt 4 | 209 | 186 | Na | 2 | | No | ND |
| Pt 14 | 8 | Na | Na | 1 | | No | ND |
| Pt 14 | 85 | 43 | Na | 2 | | No | ND |
| Pt 16 | 6 | Na | Na | 1 | | No | ND |
| Pt 16 | 98 | 58 | Na | 2 | | No | ND |
| Pt 16 | 221 | 181 | Na | 2 | | No | ND |
| Pt 17 | 72 | 44 | 10 | 3 | | No | ND |
| Pt 17 | 447 | 419 | 375 | 3 | | No | ND |
| Pt 18 | 41 | Na | Na | 1 | | No | ND |
| Pt 18 | 198 | 152 | Na | 2 | | No | ND |
| Pt 18 | 245 | 200 | 47 | 3 | | No | 15,4 |
| Pt 19 | 57 | 35 | Na | 2 | | No | ND |
| Pt 19 | 126 | 104 | Na | 2 | | No | ND |
| Pt 21 | 8 | Na | Na | 1 | | No | 16,7 |
| Pt 21 | 164 | 143 | Na | 2 | | No | ND |
| Pt 26 | before vaccine | Na | Na | 0 | | No | ND |
| Pt 26 | 131 | 55 | Na | 2 | | No | ND |
| Pt 26 | 214 | 138 | Na | 2 | | No | ND |
| Pt 26 | 249 | 173 | Na | 2 | | No | ND |
| Pt 26 | 592 | 516 | 352 | 3 | | No | ND |
| Pt 28 | before vaccine | Na | Na | 0 | low Wuhan-variant response | No | ND |
| Pt 28 | 196 | 155 | 7 | 3 | low Wuhan-variant response | No | ND |
| | | | | | | | |
| Pt 22* | 661 | 605 | 492 | 3 | | yes; ~330 days before sampling | 121,7 |
| Pt 18* | 647 | 601 | 448 | 3 | | yes; ~310 days before sampling | 53,5 |
| Pt 14* | 600 | 558 | 397 | 3 | | yes; ~270 days before sampling | ND |

***BA.1 MN titer:*** *The assay measures neuralization titer response against SARS-CoV-2/human/NOR/Bergen-05/2022 (Omicron); NCBI accession number GenBank: ON222956*

***vacc.:*** *vaccination with a delta-variant vaccine*

***d:*** *days*

***Na:*** *Not applicable*

***ND**: Not detected*

******: samples taken from three patients after the study period. At the time, these patients had both been vaccinated with a delta-variant vaccine and been infected by SARS-CoV-2 in the omicron-period*

## Figure S1

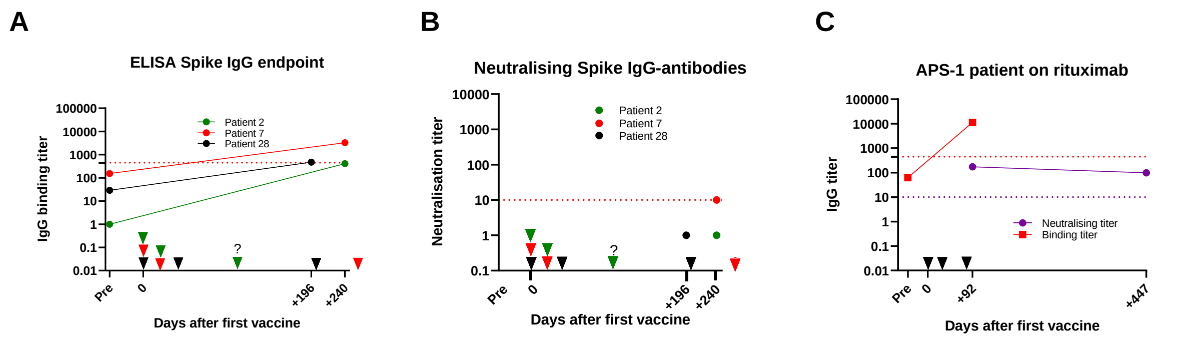


**Legends Fig. S1. A and B**. Neutralizing (**A**) (only one measurement for each patient) and binding (**B**) spike IgG antibodies in three APS-I patients with low vaccine responses. Arrows denote timepoint of vaccination. The stapled horizontal red line indicates estimated threshold required for functional response. **C.** Neutralizing (purple) and binding (red) Spike IgG antibodies in the only APS-I patient in this study that was on monoclonal immune modulating therapy. arrows denote timepoint of vaccinations. The stapled horizontal lines indicate estimated functional response thresholds for neutralization (purple) and binding (red) assays.

## Figure S2

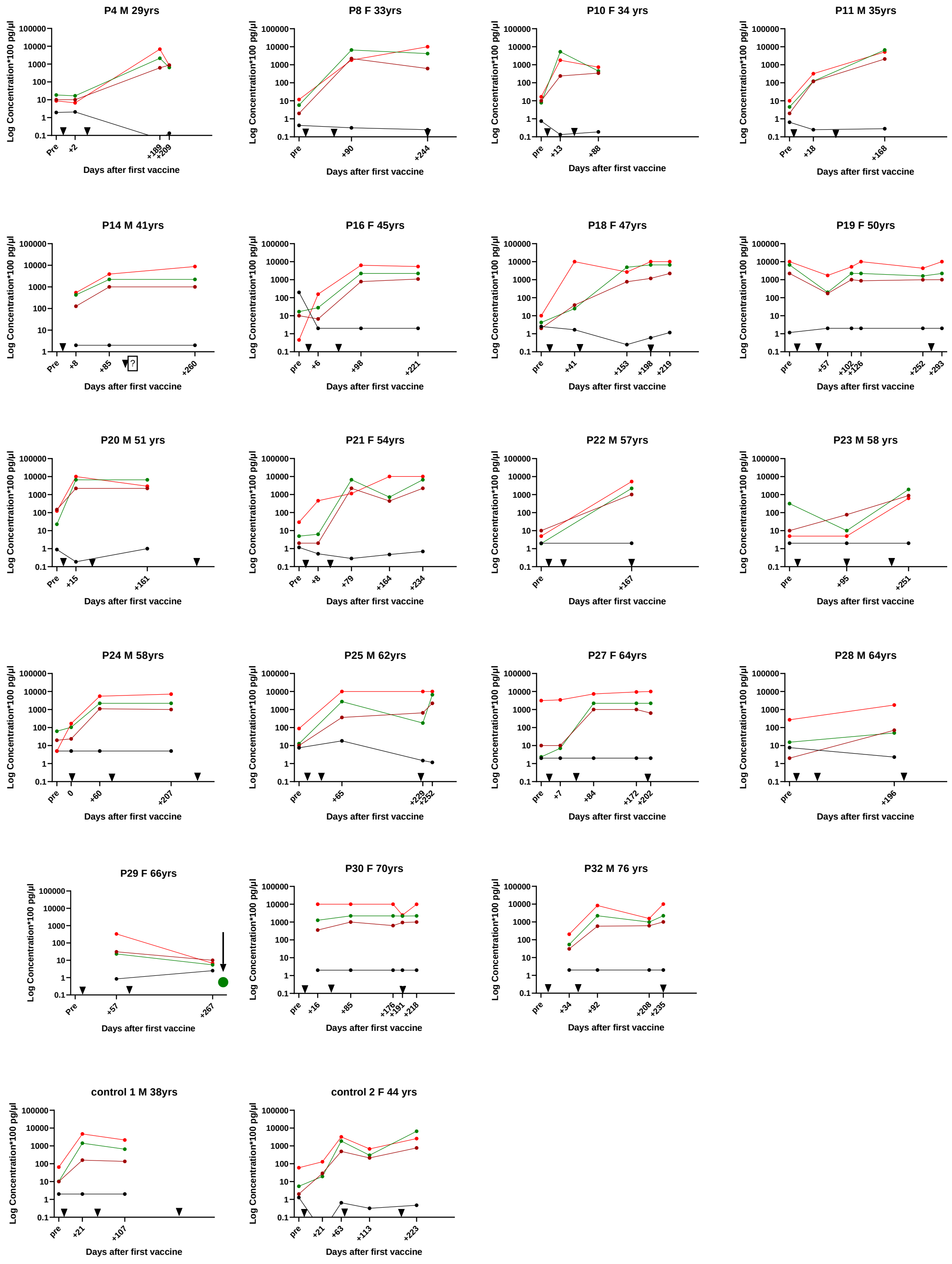


**Legends Fig. S2:** Antibody titers of longitudinal sampling of 19 APS-I patients and two healthy controls measured by a 4-plex Luminex kit. Black arrows denote timepoint of vaccinations. The graph represents responses against four SARS-CoV-2 antigens: Spike 1 (red); Spike 2 (Green); RBD (brown); Nucleocapsid (black). Arrows with a green circle denotes the timepoint of SARS-CoV-2 infection in the patients where relevant. A question mark on the x-axis denotes that the date (days after first vaccine) is not confirmed.

**Figure S3: Autoantibodies against IFN-Is in patients with APS-1 pre- and post- vaccination (N=10) with SARS-CoV-2 and after infection (N=3).**

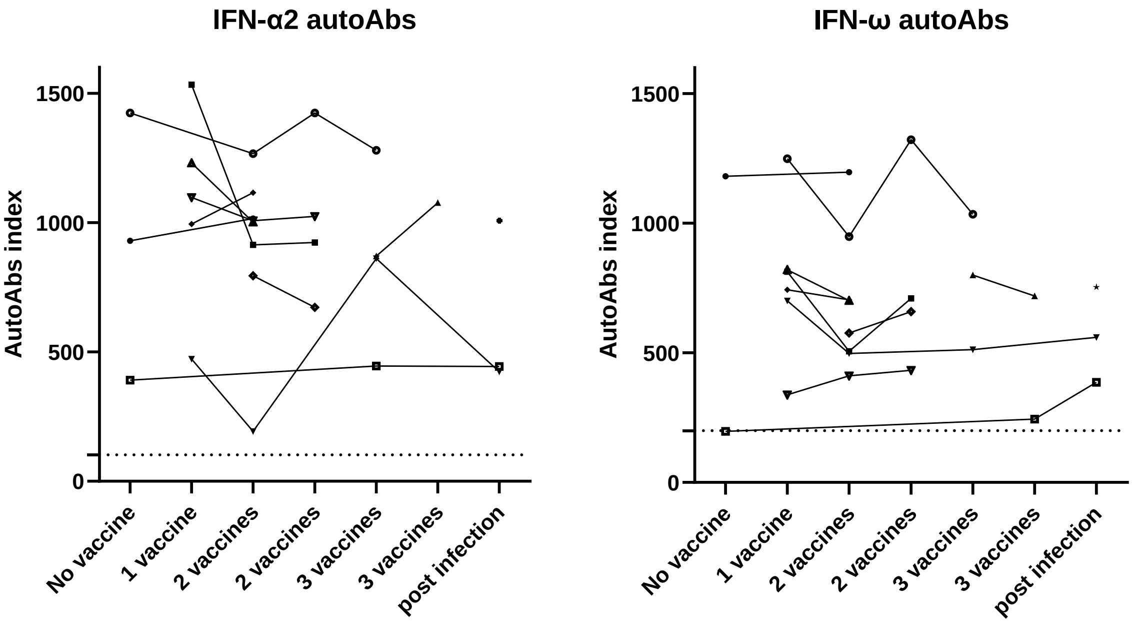


**Legends Fig. S3:** Binding autoantibodies against IFN-α2 and -ω before and after SARS-CoV-2 vaccination in 10 APS-1 patients, in addition to the response after SARS-CoV-2 Omicron-variant infections (N=3). The stapled line is the established threshold for positivity for each assay.

**Figure S4: Individual antibody responses after SARS-CoV-2 vaccination.**

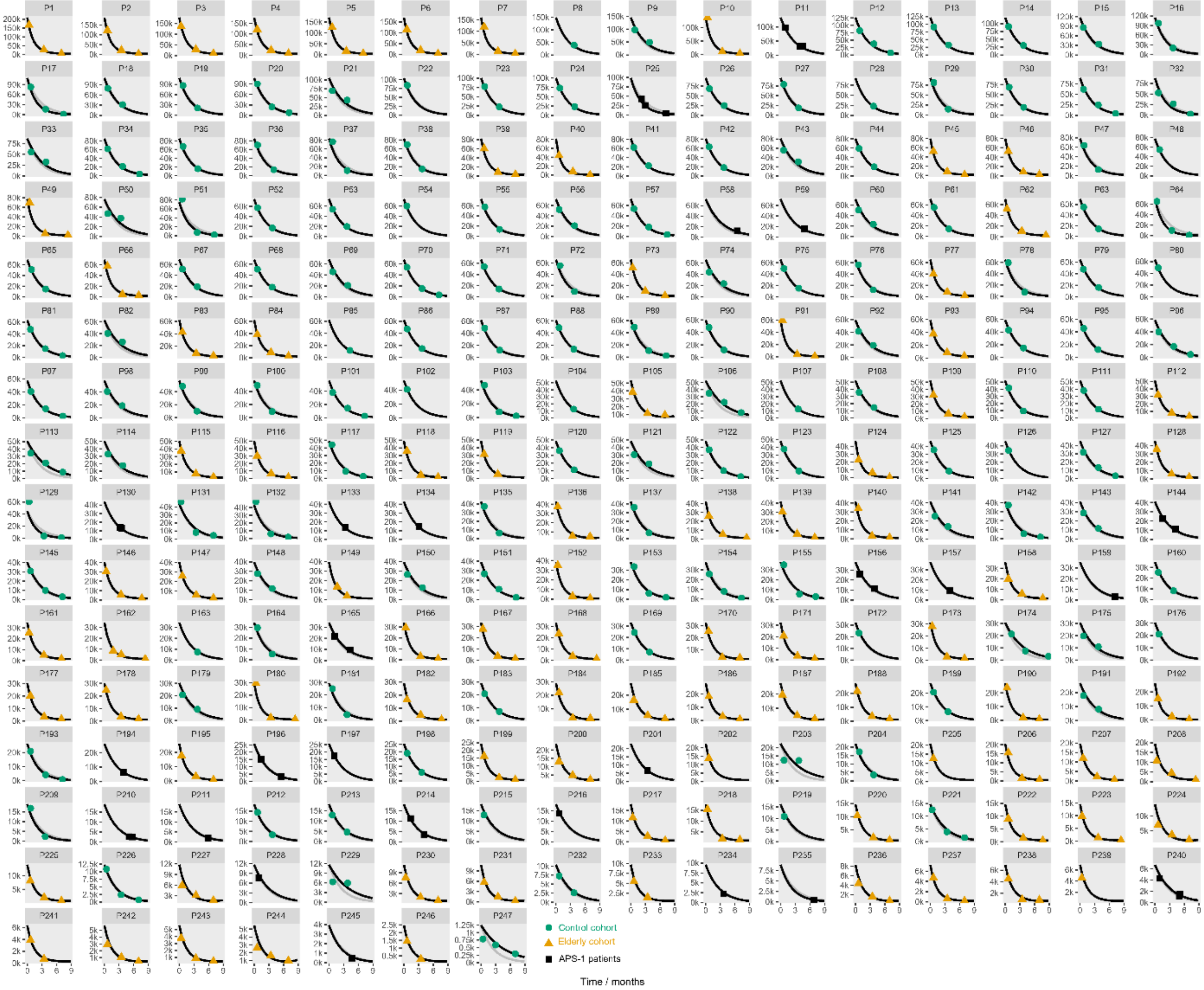


**Legends Fig S4** Measured spike IgG binding vaccine responses (colored symbols) along with model-based fitted decay curves (black curves) for each subject. APS-1 patients: black squares; matched controls: green circles; elderly cohort: orange triangles. In addition, the gray curves in each panel (only shown for APS-1 patients and their matched controls, and often obscured by the black curves) show what the fitted response would have looked like if all subjects within each group had the exact same decay rate (i.e., if there were zero variance in the random slopes). The panels have been sorted by maximum estimated vaccine response at time 0 (= 14 days after the second vaccine dose).
